# An essential role for the nuclear protein Akirin2 in mouse limb interdigital tissue regression

**DOI:** 10.1038/s41598-018-30801-2

**Published:** 2018-08-16

**Authors:** Peter J. Bosch, Leah C. Fuller, Joshua A. Weiner

**Affiliations:** Department of Biology and Iowa Neuroscience Institute, The University of Iowa, Iowa City, IA USA

## Abstract

The regulation of interdigital tissue regression requires the interplay of multiple spatiotemporally-controlled morphogen gradients to ensure proper limb formation and release of individual digits. Disruption to this process can lead to a number of limb abnormalities, including syndactyly. Akirins are highly conserved nuclear proteins that are known to interact with chromatin remodelling machinery at gene enhancers. In mammals, the analogue Akirin2 is essential for embryonic development and critical for a wide variety of roles in immune function, meiosis, myogenesis and brain development. Here we report a critical role for Akirin2 in the regulation of interdigital tissue regression in the mouse limb. Knockout of Akirin2 in limb epithelium leads to a loss of interdigital cell death and an increase in cell proliferation, resulting in retention of the interdigital web and soft-tissue syndactyly. This is associated with perdurance of *Fgf8* expression in the ectoderm overlying the interdigital space. Our study supports a mechanism whereby Akirin2 is required for the downregulation of *Fgf8* from the apical ectodermal ridge (AER) during limb development, and implies its requirement in signalling between interdigital mesenchymal cells and the AER.

## Introduction

Limb development is a complex process involving the action of specialised signalling regions that coordinate both spatially and temporally to sculpt a limb of particular shape and structure suited to a given organism. Digit formation requires the combined coordination of morphogen gradients and feedback loops that dictate responses by cells of the apical ectodermal ridge (AER), zone of polarising activity (ZPA), non-AER ectoderm, and mesenchymal cells within the limb bud^1–3^. During this process, proper gene expression changes are critical to ensure that cell proliferation and cell death are correctly balanced and confined spatially to the appropriate region of the developing limb bud. Such mechanisms lead to restriction in interdigital tissue growth and promote interdigital regression in order to produce defined and separated digits in both the forelimb and hindlimb. Disruption in morphogen release, receptor-mediated responses, or changes in cell proliferation and cell death can lead to many limb abnormalities, including soft-tissue syndactyly (fused/webbed digits)^4–7^.

Akirins are highly conserved, small nuclear proteins that have been shown to localise to promoter and enhancer regions of genes, despite a lack of apparent DNA-binding domains^8,9^. Current evidence suggests that Akirins function as “bridge” proteins that interact with transcription factors and chromatin remodelling machinery to coordinate a vast array of gene expression patterns. *Drosophila* and *C*. *elegans* have a single *Akirin* gene, whereas mammals have two homologues, *Akirin1* and *Akirin2*^10^. Complete null mutants for *Akirin1* are viable and outwardly normal; however, *Akirin2* null embryos were not found on embryonic (E) day 9.5, indicating that it is essential for early embryonic development^11^. Because of this, transgenic mouse models produced by crossing a conditional floxed allele of *Akirin2*^11^ to *Cre* transgenics are required to selectively restrict *Akirin2* deletion to discrete populations of cells in a given tissue of interest.

Recently, we identified an essential role for Akirin2 in the formation of the cerebral cortex and hippocampus, utilising *Emx1-Cre* to excise the *Akirin2* gene from the developing telencephalon^12^. Mutant embryos displayed near-agenesis of the cortex due to early cell-cycle exit and subsequent apoptosis of telencephalic progenitor cell and nascent neuron populations. To date, important roles have also been reported for Akirins in myogenesis, meiosis, immune function, and gene regulation in *Drosophila*, *C*. *elegans*, *Xenopus* and mammals^9,13–16^. Akirin2 has been shown to have an important functional role in the nucleus, acting as a bridge protein that binds to Brg1-associated factor 60 (BAF60a/b/c) and IκB-ζ to allow binding and activation of the IL-6 promoter^8^. Akirin2 is also important for the recruitment of the BAF complex core helicase Brg1 to *Myc* and *Cyclin D2* promoters^17^. Akirin- (*Drosophila*) or Akirin2- (mouse) dependent genes include those whose promoters have a low number of CpG islands, consistent with an association of Akirins with chromatin remodelling machinery^8,18^. Additionally, *Drosophila* Akirin localisation corresponds with the active transcriptional mark, acetylated H3K9, further demonstrating a role in gene transcription^18^. Akirin has been shown to be important for muscle development in *Drosophila*, with mutants displaying diverse phenotypes including absent, misattached, or duplicate muscles^9^, and recent *in vitro* studies also suggest a role for Akirin2 in porcine muscle cell proliferation^19^. In the course of our studies^12^ on *Akirin2* in the developing brain using *Emx1-Cre*, which is also active in limb epithelium^20^, we have uncovered *in vivo* evidence that Akirin2 critically regulates the regression of interdigital tissues in the developing limb bud.

Following a period of limb outgrowth that initiates with the formation of a limb bud from lateral plate mesoderm, individual digits are formed using a tightly regulated process of programmed cell death (PCD). At around embryonic day (E) 12.5 in mice, BMP2 and BMP4 released from the interdigital mesenchyme underlying the limb ectoderm activate BMP receptor/Smad signalling in the AER, a pseudostratified epithelium that rims the dorsal-ventral border of the distal limb bud^21,22^. The AER is an FGF-rich signalling center essential to support limb bud outgrowth; FGF8 is the only FGF continually expressed during this time, although FGF4, FGF9 and FGF17 are also expressed at various times in the AER^21^. Proper BMP signalling and subsequent downregulation of FGF expression initiates PCD in the interdigital mesenchyme cells, which allows for separation of individual digits between E13-E14.5^23^.

Developmental disruption of interdigital tissue regression leads to soft tissue syndactyly (i.e., tissue fusion without bone fusion), a human congenital defect that occurs in ~1 in 2000 live births^24^. Syndactyly typically occurs concurrent with reduced apoptotic markers and continued proliferation of cells in the interdigital space^5,7,25,26^. Mechanistically, soft-tissue syndactyly appears to be caused primarily by prolonged FGF signalling, providing a cell-survival signal that allows interdigital cells to escape PCD: FGF4 overexpression in limb ectoderm leads to persistent interdigital tissue^27^, FGF8-soaked beads reduce the number of dying interdigital cells^28^, and enduring *Fgf4* and *Fgf8* expression is seen concurrent with soft-tissue syndactyly^5,7^. Prolonged FGF expression is consistently seen during experiments designed to impair BMP2/BMP4 function in the mesenchyme. For example, interdigital tissue regression is impaired when *Bmp2/4* knockout is restricted to the limb bud^29^, when dominant-negative BMP receptors are expressed in the limb bud^30^, or when knockout of both *Bmpr1a*^5^ and *Smad1/5* are restricted to the AER^7^. Overall, these results suggest that experimental loss of Smad signalling likely prevents BMP from downregulating FGFs, resulting in the continued cell proliferation and cell survival of interdigital tissue^5,7^.

Here, we show that Akirin2 is essential for interdigital tissue regression in the limb *in vivo*. Loss of Akirin2 in the limb ectoderm (using *Emx1-Cre*) results in a persistent soft-tissue syndactyly due to a lack of PCD and continued cell proliferation in interdigital tissues. This appears to be caused by aberrantly persistent *Fgf8* expression in the AER cells adjacent to the fused digits. Our data demonstrate a previously-unsuspected role for Akirin2 in interdigital tissue regression and imply its requirement for BMP signalling and/or subsequent downregulation of *Fgf8*.

## Results

### Akirin2 is expressed in the developing limb

**Figure 1:**
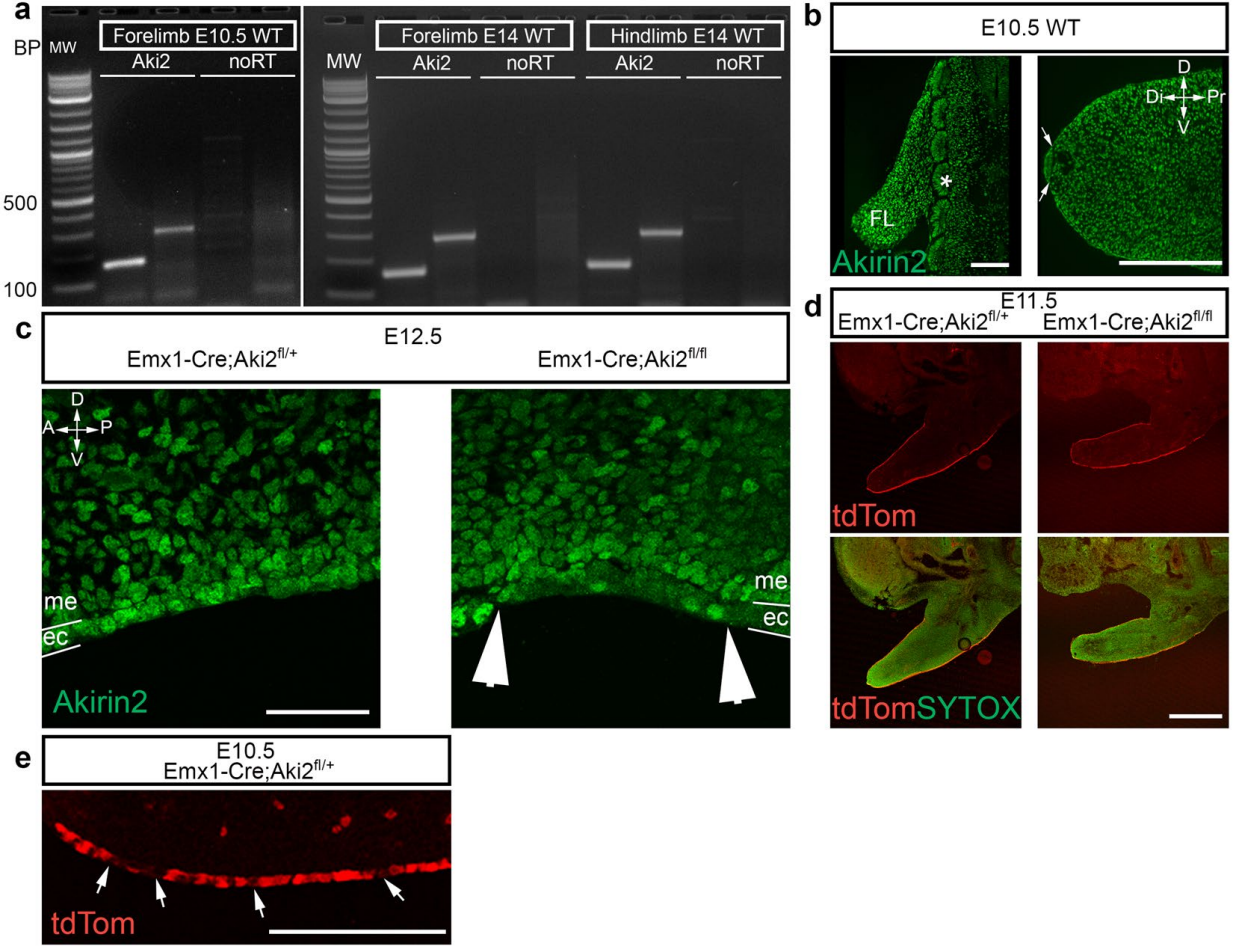
Akirin2 expression in the developing limb bud epithelium. (**a**) RT-PCR using two different primer sets that generate amplicons across multiple exons. *Akirin2* is expressed in the limb at E10.5 and E14. (**b**) In wild type E10.5 cryosections, strong Akirin2 immunoreactivity is seen in the somites (denoted by *) and in the forelimb bud (FL), including expression in the AER (arrows). (**c**) At E12.5, Akirin2 immunostaining is lost in most cells of the ectoderm overlying the interdigital tissue of the knockout forelimb compared with control; as expected, mesodermal expression is unaffected as Cre is limited to the ectoderm. (**d**) Expression of a tdTom Cre reporter allele in the developing limb at E11.5 in both control and mutants, counterstained with the nuclear marker Sytox Green, shows epithelium-restricted Cre activity. (**e**) High magnification image of the tdTom reporter showing widespread, but patchy, expression in the ventral ectoderm of E10.5 control forelimb (arrows indicate tdTom-negative cells). Key: A, anterior; Di, distal; D, dorsal; ec, ectoderm; FL, forelimb; me, mesoderm; P, posterior; Pr, proximal; V, ventral. Scale bar: 100 μm in b; 50 μm in c; 200 μm in d; 50 μm in e.

Akirin2 expression has been reported in multiple rodent and human tissues, including heart, lung, testis, spleen, liver, intestine, placenta, ovary, thymus, kidney, muscle, blood leukocytes, and brain^11,12,15^. Reverse-transcription (RT)-PCR demonstrated the presence of *Akirin2* transcripts in limb buds at E10.5 and E14 (Fig. 1a) and immunostaining using an Akirin2-specific antibody confirmed protein expression in the forelimb bud and somites at E10.5 (Fig. 1b), as well as robust expression in the AER (Fig. 1b, between arrows). Further staining confirmed Akirin2 protein expression in both ectodermal and mesenchymal limb bud cells at E12.5 (Fig. 1c). *Emx1* is expressed in the developing limb epithelial AER^20^, and in the course of our studies on *Akirin2* functions in cerebral cortex development^12^, we noted widespread, *Emx1-Cre*-mediated activity via a tdTomato (tdTom) reporter allele (*Ai14-tdTomato*) in the embryonic limb epithelium (Fig. 1d). Close examination of tdTom signal in the epithelium revealed that Cre activity was somewhat patchy, with interspersed negative cells (Fig. 1e; see also below). Demonstrating antibody specificity, in *Emx1-Cre;Akirin2*^*fl/fl*^ (hereafter referred to as Emx-KO) limb buds, Akirin2 staining was eliminated from many cells of the epithelium, but remained unaffected in the Cre-negative interdigital mesenchyme (Fig. 1c, between arrowheads). As we reported previously^12^, most Emx-KO mice died shortly after birth due, presumably, to a near-complete absence of the cerebral cortex, with a small minority surviving into late adolescence.

### Loss of Akirin2 in limb bud epithelium leads to soft-tissue syndactyly

**Figure 2:**
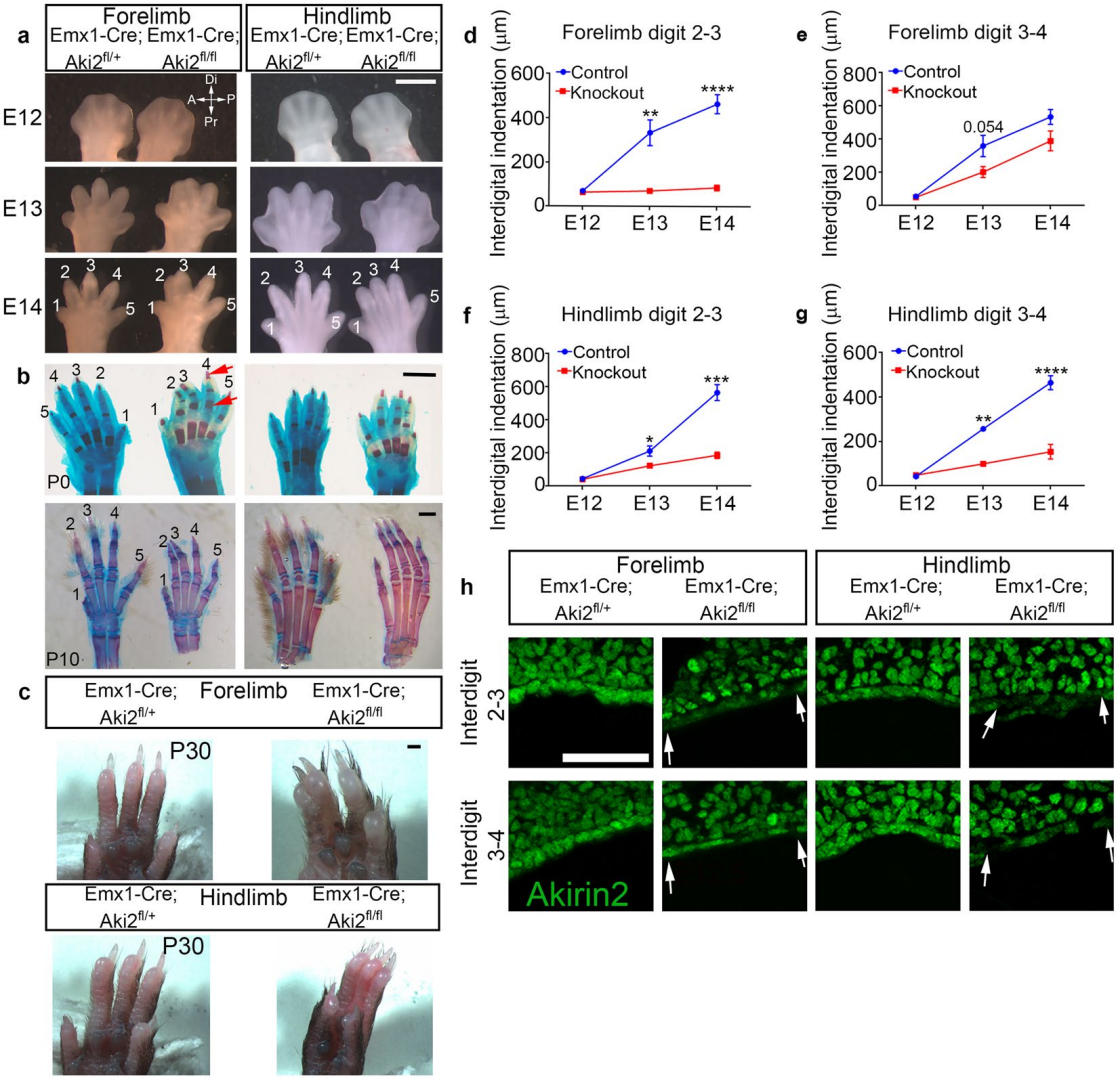
Loss of Akirin2 in the limb epithelium leads to soft-tissue syndactyly. (**a**) Interdigital tissue regression is impaired in *Akirin2* Emx-KO forelimb (digits 2/3) and hindlimb (digits 2/3/4). (**b**) Alcian Blue/Alizarin Red staining at P0 and in one of the few surviving mutants at P10 shows that the *Akirin2* Emx-KO limb does not have fused bone or cartilage elements, though broader Alizarin Red (bone) staining is observed in the phalanges (red arrows). (**c**) A very rare *Akirin2* Emx-KO that survived until P30 contains clear syndactyly of the mature forelimb and hindlimb. (**d**–**g**) Quantification of interdigital tissue regression shows impaired tissue regression between digits 2–3 of the forelimb (**d**) and 2–4 of the hindlimb (**f**,**g**) in *Akirin2* Emx-KO mice. The difference in forelimb interdigital regression between digits 3–4 approaches statistical significance (**e**; p = 0.054). n = 3 animals/genotype for E12 & E13; 4 animals/genotype for E14, *p < 0.05, **p < 0.01, ***p < 0.001, ****p < 0.0001, t-test. (**h**) Immunostaining of *Akirin2* Emx-KO distal limb buds at E12.5 shows that Akirin2 expression is extensively lost in the interdigital space between digits 2–3 in both fore- and hindlimb. In the forelimb, most cells between digits 3–4 remain Akirin2-positive, while in the hindlimb more Akirin2-negative cells are observed (interdigital regions indicated between arrowheads). Scale bar: 1 mm in a-c; 50 μm in h.

With complete penetrance, *Emx1-Cre-*restricted loss of *Akirin2* from limb bud epithelia led to a syndactyly phenotype that differed between fore- and hind-limbs (Fig. 2). In the forelimb, digits 2 and 3 remained fused, while in the hindlimb, digits 2, 3 and 4 remained fused (Fig. 2a–c). To determine whether this was a soft-tissue syndactyly or bone fusion was involved, we stained limbs with Alcian Blue (cartilage) and Alizarin Red (bone) at P0 and P10 (Fig. 2b). There was no clear fusion of bone or cartilage elements; however, it was clear that interdigital tissue was retained between the adjacent digits. There also appeared to be an increased area of Alizarin Red staining in the phalanges of Emx-KO mice (red arrows, Fig. 2b), similar to that observed following *Bmpr1a* knockout in the AER^5^. Soft-tissue syndactyly was maintained into postnatal development, as the few surviving Emx-KO mice still exhibited fused digits at P10 (Fig. 2b) and P30 (Fig. 2c).

To track the development of this syndactyly, we quantified interdigital tissue regression in control and Emx-KO mice (Fig. 2d–g). Whereas in control forelimbs interdigital tissue steadily regressed between E12 and E14, in the forelimb of Emx-KO mutants interdigital tissue remained unchanged between digits 2–3 and was somewhat slower to regress between digits 3–4 (Fig. 2d,e). In the mutant hindlimb, interdigital tissue regression was minimal between digits 2–3 and 3–4 between E12 to E14 (Fig. 2f,g). These embryonic measurements are fully consistent with the syndactyly phenotypes observed at embryonic, perinatal, and postnatal ages (Fig. 2a–c). Previous work^31^ and our own observations (Fig. 1c–e) indicate that Emx1-Cre activity is widespread but patchy in the limb bud epithelium. Because Akirin2 expression is uniform in the limb epithelium (Fig. 1b,c), we thus asked whether patchy Cre activity underlies the observed differences in interdigital regression between forelimbs and hindlimbs. We immunostained cryosections from E12.5 forelimbs and hindlimbs with an Akirin2 antibody. Indeed, careful inspection of the distal limb bud tips revealed that in Emx-KO forelimbs, Akirin2 immunoreactivity is nearly completely absent from ectodermal cells between digits 2–3, but most cells retain Akirin2 between digits 3–4. In contrast, in the hindlimb, extensive Akirin2 loss was identified in the interdigital tissue between both digits 2–3 and 3–4 (Fig. 2h). This indicates that the expression of *Emx1-Cre* differs between forelimb and hindlimb ectoderm, and that localized loss of Akirin2 can account for the pattern of resulting syndactyly that we observe.

### Akirin2 knockout leads to reduced PCD and continued proliferation of interdigital tissue

**Figure 3:**
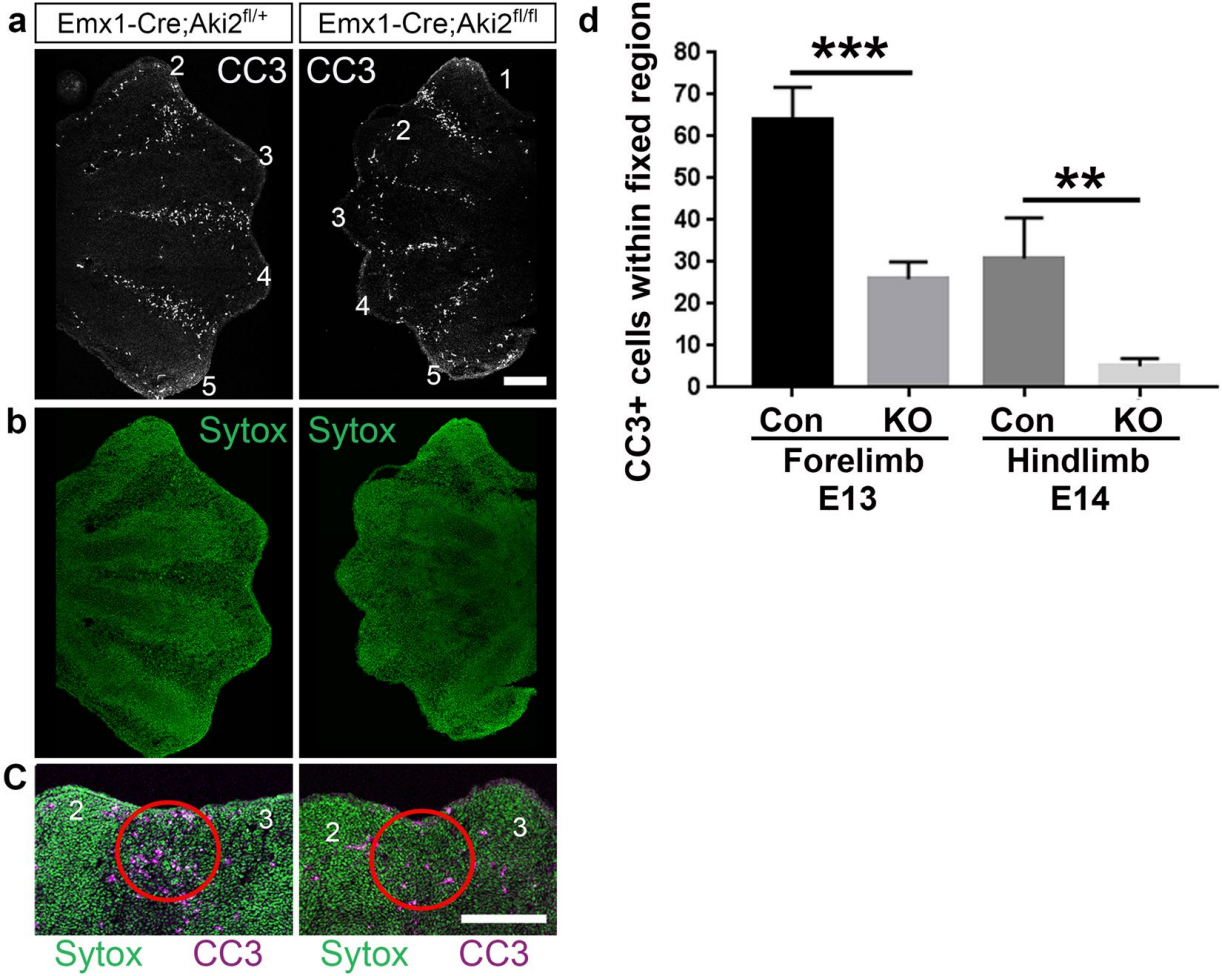
Akirin2 loss in limb epithelium leads to decreased programmed cell death between digits that display syndactyly. (**a**) Cleaved caspase-3 staining is nearly absent between digits 2–3 in *Akirin2* Emx-KO forelimb and reduced between the other digits. (**b**) Sytox Green cellular stain of the limb sections in (**a**). (**c**) High magnification views of the distal limb bud region between digits 2 and 3 demonstrates reduced cleaved caspase-3 staining in the Emx-KO interdigital tissue (red circle). (**d**) Quantification of the number of cleaved caspase-3 cells within the fixed region outlined in (**c**; Con = Control). Key: CC3, cleaved caspase-3; n = forelimb E13: Con 21 sections, 5 animals; KO 19 sections, 5 animals; hindlimb E14: Con 14 sections, 3 animals; KO 18 sections, 3 animals. **p < 0.01, ***p < 0.001, t-test. Scale bar: 200 μm.

During normal murine limb development, cells in the interdigital tissue begin to undergo PCD around E13.5 (forelimb) and E14 (hindlimb), allowing tissue regression and the release of individual digits^23^. Reduced or absent cell death in the interdigital space has been shown by multiple studies to result in syndactyly^5–7,25,27^. We thus asked whether the disrupted regression of interdigital tissue and resultant syndactyly in Emx-KO mutants were due to impaired PCD. We stained E13 forelimb and E14 hindlimb cryostat sections with antibodies against the apoptotic marker, cleaved caspase-3 (CC3) and quantified CC3-positive cells. Indeed, reduced CC3 staining corresponded with the location of digits that displayed syndactyly (Fig. 3a,b), and this was especially apparent at the distal tip of the interdigital tissue (Fig. 3c). Quantification of CC3-positive cells in a fixed region at the distal tip of the interdigital tissue showed that it was significantly reduced in Emx-KO mice compared to controls at E13 in the forelimb and E14 in the hindlimb (hindlimb development normally lags somewhat behind forelimb^3,23^) (Fig. 3d). Interestingly, given the massive PCD observed in *Akirin2*-null neural progenitors^12^, no increase in CC3 staining, was observed in Akirin2-null epithelium itself (Fig. 3c). As mesenchymal limb tissue retains Akirin2 expression in Emx-KO mutants (Fig. 1c), the disruption of interdigital tissue PCD thus represented a cell-non-autonomous phenotype.

**Figure 4:**
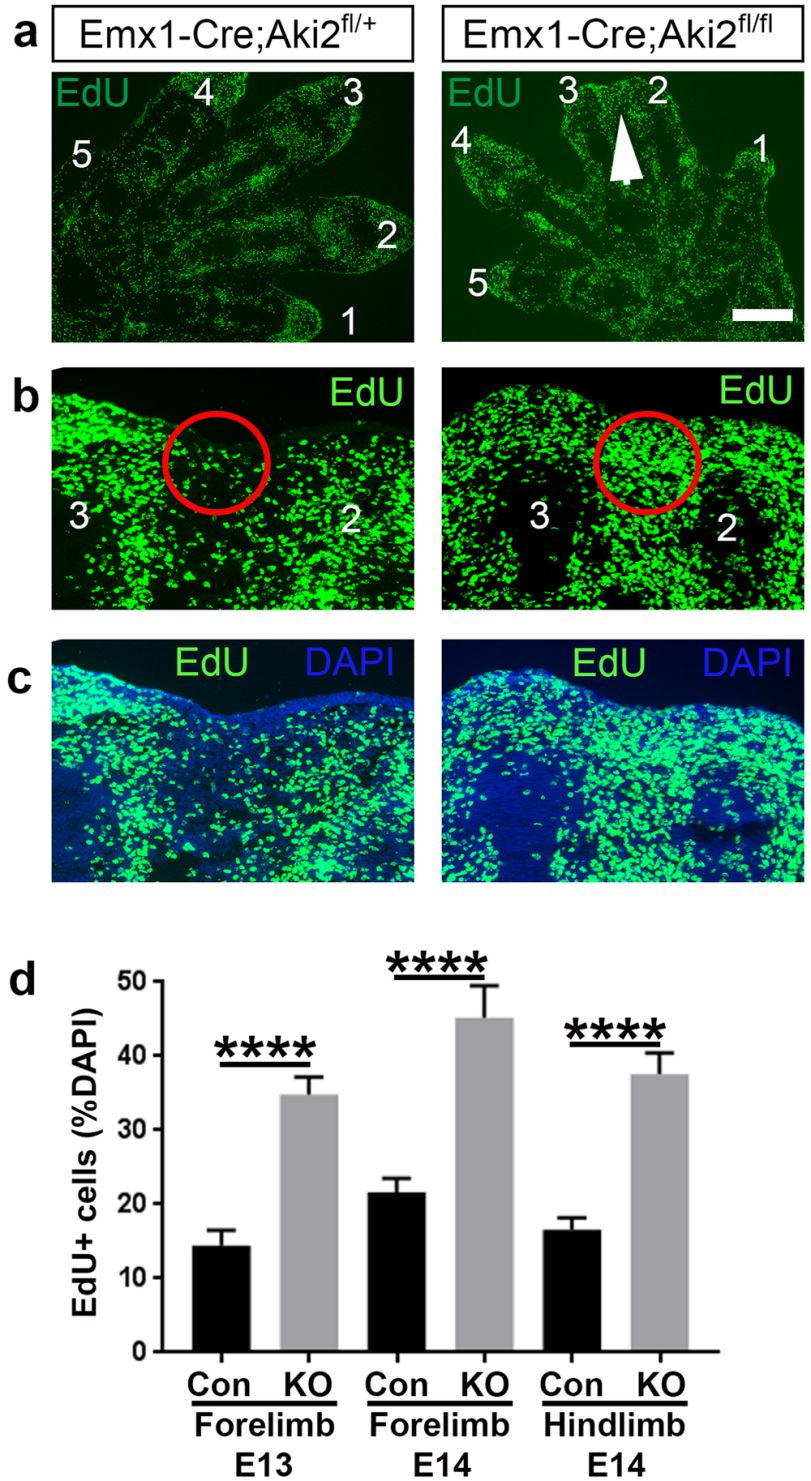
Akirin2 loss in limb epithelium leads to increased cell proliferation between digits that display syndactyly. (**a**–**c**) Immunohistochemistry of EdU with DAPI counterstain shows increased EdU-positive cell density at the distal end of the limb at E13 and E14 (**a**, low magnification, white arrowhead; and **b**,**c**, high magnification, red circles; digits are numbered). (**d**) Quantification of percent DAPI-positive cells that are also EdU-positive in control and *Akirin2* Emx-KO in the E13 forelimb and E14 forelimb and hindlimb. Cell counts were made in the distal interdigital areas, shown outlined by the red circles. n = forelimb E13: Con 21 sections, 3 animals; KO 25 sections, 4 animals; forelimb E14: Con 17 sections, 3 animals; KO 18 sections from 3 animals; hindlimb E14: Con 18 sections, 3 animals; KO 18 sections, 3 animals. ****p < 0.0001, t-test. Scale bar (in a): 300 μm in a; 100 μm in b,c.

Reduced PCD suggested that there may be continued cell proliferation in the interdigital tissue of Emx-KO embryos. To investigate this, we injected the nucleotide analogue EdU into pregnant dams at E13 and E14, collected embryos 2 hours later, and stained cryostat sections for EdU uptake, indicative of cell proliferation. In the Emx-KO mutant limb, we found an increase in EdU-positive proliferating cells within the interdigital tissue; again, this was especially apparent at the distal tip (Fig. 4a–c). The region of the limb with increased numbers of proliferative cells coincided well with the observation of syndactyly (Fig. 4a). At E13 in the forelimb, and at E14 in both the forelimb and hindlimb (Fig. 4d), interdigital tissue contained significantly higher densities of EdU-positive cells (normalised to total cell numbers). Together, these data indicate that the syndactyly phenotype in Emx-KO mice is caused by aberrant continued survival and proliferation of interdigital tissues.

### Aberrantly retained Fgf8 signal in Akirin2 knockout interdigital ectoderm

**Figure 5:**
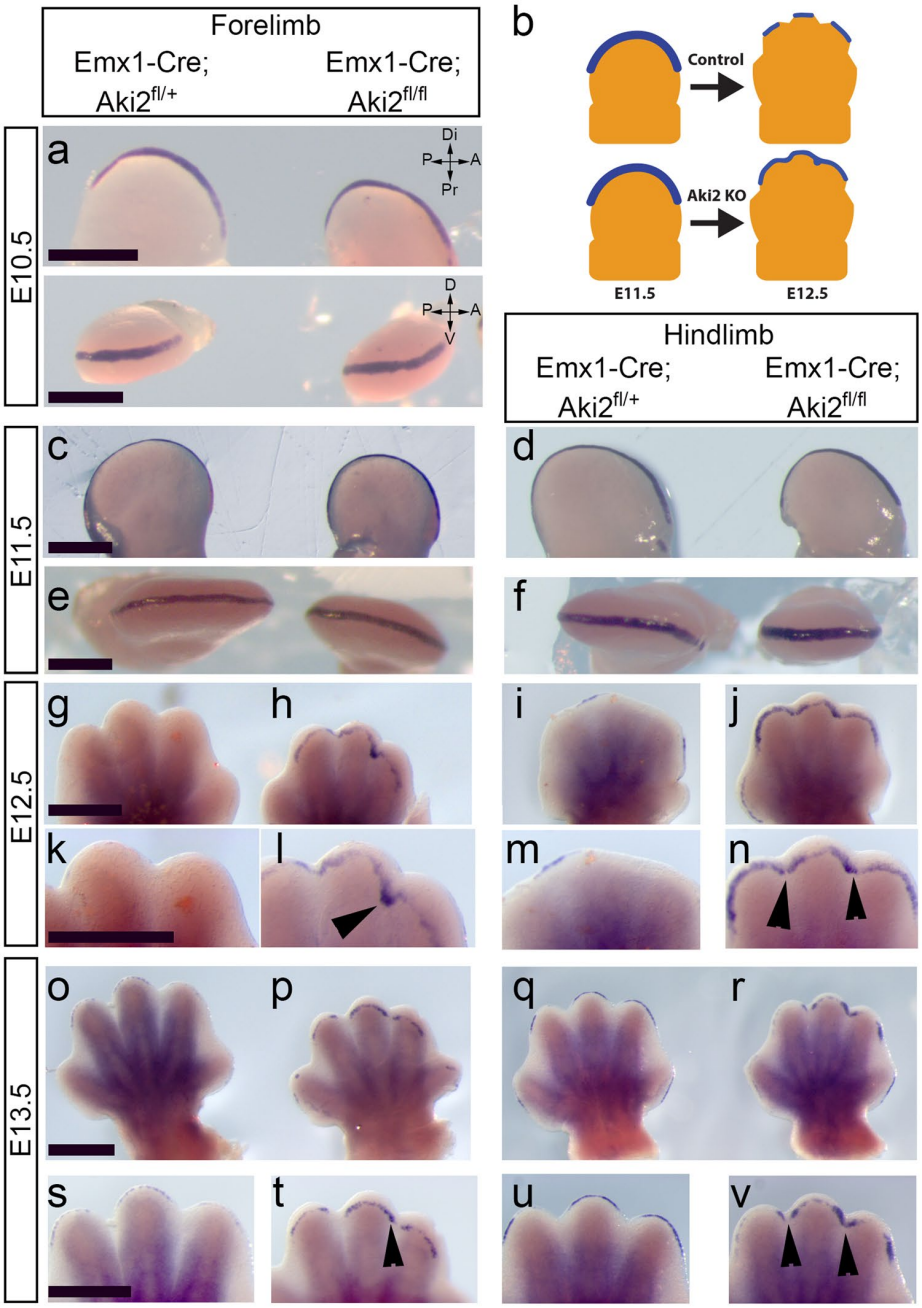
*Fgf8* expression is aberrantly retained in *Akirin2* knockout limb epithelium. Whole mount *in situ* hybridisation using *Fgf8* riboprobes comparing control and *Akirin2* Emx-KO limb at E10.5 (**a**), E11.5 (**c**–**f**), E12.5 (**g**–**n**) and E13 (**o**–**v**). *Fgf8* signal is retained in the *Akirin2* Emx-KO limb distal epithelia in the regions that display syndactyly from E12.5 onwards (**g**–**v**): digits 2–3 in forelimb and digits 2–4 in hindlimb (black arrowheads). Insets at E12.5 (**k**–**n**) and E13 (**s**–**v**) show higher magnification images of the distal limb. Panel (b) is a schematic showing the KO phenotype of perduring *Fgf8* expression (blue). Key: A, anterior; D, dorsal; Di, distal; P, posterior; Pr, proximal; V, ventral. Scale bar: 500 μm in each row.

The initiation of interdigital tissue regression is thought to involve BMP ligand release from the mesenchyme underlying the AER, and subsequent attenuation of FGF8 signalling within the AER^5^. Because Emx-KO embryos lack *Akirin2* in the limb epithelium (including the AER), but not the mesenchyme, we reasoned that FGF8 loss in the AER might be disrupted. Whole mount *in situ* hybridisation for *Fgf8* showed clear differences in expression between control and mutant forelimbs and hindlimbs (Fig. 5). Between E10.5 and E11.5, we found no apparent difference in the extent or intensity of *Fgf8* signal between controls and mutants (Fig. 5a–f). From E12.5 on, however, it became clear that *Fgf8* signal aberrantly perdured in the AER overlying the interdigital mesenchyme in Emx-KO forelimb and hindlimb (Fig. 5g–j and o-r, low magnification images; Fig. 5k–n and s-v, higher magnification images). The regions of the AER in which *Fgf8* signal was aberrantly retained corresponds exactly with where reduced PCD and increased cell proliferation were observed in the underlying mesenchyme (Figs 3, 4). We also performed quantitative RT-PCR (qPCR) analysis of E12.5 forelimbs, investigating the expression of an array of genes that are known to influence interdigital development (Supplementary Fig. S1). We found no significant difference in the expression levels of any genes examined, which suggests that Akirin2 may act directly on the expression of *Fgf8* at a chromatin level, rather than by affecting BMP receptor expression or by affecting (cell non-autonomously) mesenchymal release of BMPs. We conclude that Akirin2 is required in the developing limb bud epithelium for the downregulation of *Fgf8* that triggers interdigital tissue regression and the separation of digits.

## Discussion

In this study we restricted *Akirin2* gene knockout to the limb ectoderm using *Emx1-Cre*, and demonstrated a role for Akirin2 in interdigital tissue regression for the first time, identifying a novel nuclear effector within the ectoderm that is critical for this process. Akirin2 is expressed throughout the developing limb bud, in both ectoderm and mesoderm, and loss of *Akirin2* from the ectoderm leads to soft-tissue syndactyly. This is due to reduced PCD and enduring cell proliferation in the relevant digits, and continued *Fgf8* expression in these digits beyond its normal physiological timeframe.

Recent studies propose a model of interdigital web regression whereby BMP2/4 release from the interdigital region of the limb activates BMPR1a in the AER, which signals through Smads, resulting in downregulation of FGF8. The importance of BMPs is shown by studies in which high levels of the BMP-inhibitor, *Gremlin*, were reported in the interdigital tissue of naturally-occurring webbed limbs such as the bat wing^32^ and duck hindlimb^33^ during development. BMP signalling thus contributes to removal of the cell survival signal (i.e., FGF8) for interdigital mesenchymal cells and leads to PCD^5^. In this study, we showed reduced PCD and increased proliferation in the interdigital tissue of Emx-KO limbs, similar to previous reports of syndactyly phenotypes^7,34^, implying the survival of interdigital cells that are normally fated to die. Alterations in the balance of PCD and cell proliferation in the interdigital tissue have been linked to syndactyly, and interdigital mesenchyme cell survival has been repeatedly linked to retention of FGF expression in the AER. More specifically, when FGF8 is not appropriately downregulated at the onset of interdigital web regression, syndactylies occur^5,7,25^. Impaired BMP signalling in syndactyly models is observed concurrent with enduring FGF8 expression within a persistent AER, which in normal limbs is progressively lost via PCD starting at E12.5, initially at the interdigit and then at the tips of the digits^35^. We demonstrated that *Fgf8* remains high specifically in the ectoderm overlying the interdigital spaces of fused digits in Emx-KO mutant mice.

Interestingly, at E11.5 there was no large expansion of the AER in the dorsal-ventral axis, unlike that found in previous work that impaired BMP or Smad signalling^5,6,25^, suggesting that AER maturation occurred normally in the absence of Akirin2. Furthermore, using qPCR of forelimbs at E12.5, we did not detect any change in multiple *Bmps*, *Fgf4*, their receptors, or other genes known to influence web regression. It should be recalled that these qPCR results are from whole limb buds, and thus provide an overview of gene expression in the (wild-type) mesenchyme and (Akirin2-null) ectoderm combined. However, we also appreciate the limitations of such an approach. Specifically, any localized changes that may have occurred within the ectoderm of Emx-KO mutants are unlikely to have been detected. Nevertheless, as Bmps are expressed highly in the mesenchymal limb tissue, which made up the majority of the cDNA samples analyzed in our assay, we would likely have been able to detect changes in Bmp gene expression in Emx-KO, had any occurred. A number of observations in this study suggest that the role of Akirin2 in the ectoderm is to aid in the downregulation of *Fgf8* at the onset of interdigital regression: 1) we detected no changes in *Bmp* or *Shh* gene expression in E12.5 mutant limbs; 2) the *Fgf8* expression field is only different between control and mutants at the timepoint when it normally diminishes (i.e. E12.5); and 3) in this model *Akirin2* is knocked out only in the ectoderm, so cannot have a cell-autonomous effect on interdigital mesenchymal cells. Our results also further strengthen the hypothesis that FGF8 loss is the primary signal that induces interdigital web regression^4,5,28^. We have, however, not yet provided evidence that Akirin2 directly regulates FGF8 expression, either by binding to the *Fgf8* promoter/enhancer, or by altering upstream effectors. Future studies, for example utilising chromatin immunoprecipitation with an appropriate Akirin2 antibody, will be important for elucidating the role of Akirin2 in the nucleus of ectoderm cells during interdigital regression.

The most well-established mechanism leading to syndactyly proposes that the activation of BMPR1a and Smad signalling in the AER are likely to initiate interdigital tissue regression^5,7,36^. To support this, a dominant negative form of BMPR1a expressed in the limb bud inhibited PCD in the interdigital tissue^30^ and *BMPR1a* conditional knockout in the AER caused retained *Fgf8* signal, reduced PCD and syndactyly^5^. In addition, the overexpression of BMP inhibitors Noggin^36^ and Gremlin^33^ severely reduce PCD in the interdigital tissue of limbs, and ectopic expression of Noggin in the AER to inhibit AER-BMPs leads to syndactyly and expansion of the *Fgf8* expression domain^6^. Furthermore, the double-inactivation of *Smad1/Smad5* from the AER during limb development caused impaired web regression and resulted in syndactyly, likely due to the inability to transmit BMP2/4 signals to the nucleus^7^. Despite this progress, the precise nuclear events that surround this proposed role at the chromatin level are currently unclear.

Smads are DNA-binding molecules that can activate or inhibit gene expression and have roles throughout embryonic development. Canonical BMP signalling functions through the activation of phosphorylated Smad1/5/8, which subsequently interacts with Smad4, and this complex alters gene expression in the nucleus (for detailed review, see^37^). Due to the relatively weak affinity that Smads have for DNA, additional proteins such as DNA-binding factors, coactivators and corepressors are required and recruited to promoter sites to strengthen interactions and thus change gene expression^37^. Thus, a possible role for Akirin2 during interdigital regression is via the organisation and dynamic remodelling of chromatin during Smad signalling in the ectoderm.

Analysis of the *Fgf8* gene has found that the promoter region contains both NFκB and retinoic acid receptor (RAR) binding elements (RAREs)^38,39^. The interaction of Akirin2 with NFκB signalling systems is currently its most well-documented^8,11,18^; however, to date there is limited information about the role of NFκB in syndactyly. Interestingly, a comparative proteomics study that assessed expression profiles of E12.5 and E13.5 hindlimb interdigital tissue found upregulation of protein disulfide isomerase (PDI) and downregulation of peroxiredoxin-1 (Prdx1) at a stage where interdigital cells are irreversibly committed to PCD^40^. Subsequent transfection of siRNA against Prdx1 into interdigital cells in culture found upregulation of NFκB, and the authors suggest that this is a response to high levels of reactive oxygen species in the interdigital cells at that stage of development. However, here we removed Akirin2 from the ectoderm and not the interdigital mesenchyme cells, which retain Akirin2 protein expression (Fig. 1c); thus our results do not support an interaction between Akirin2 and NFκB signalling as the cause of the syndactyly that we observe.

RARs have been shown to suppress *Fgf8* expression in various tissues, including ectoderm^39,41^. In addition, retinoic acid-soaked beads transplanted into the anterior heart field of chick embryos strongly downregulated the expression of *Fgf8*^42^. Our results indicate that Akirin2 is important for the repression of the *Fgf8* gene. Given the presence of RAREs in the *Fgf8* promoter^39^, one possible mechanism of Akirin2 function in the ectoderm would be interaction with RARs. Retinoic acid may act to limit the spread of *Fgf8* to a restricted zone during limb bud initiation (~E9.5)^43^, though loss of retinoic acid in the limb bud does not alter the *Fgf8* expression field at E10.5^44^. Nevertheless, limbs lacking retinoic acid later exhibit interdigital webbing^44^, and compound mutants of RARα/RARγ exhibit syndactyly^45^. Together, these phenotypes are consistent with what we observe in Akirin2 mutants: normal limb patterning; lack of change in *Bmp2/4*; and no dorsal-ventral expansion of the *Fgf8* expression field at E10.5 and E11.5. It is important to note; however, that retinoic acid-soaked mouse limb buds were shown to have unaltered *Fgf8* expression^28^ and mouse hindlimbs that were examined following retinoic acid loss via *Raldh2* knockout also display no change in *Fgf8* at E13.5^46^. These last two results are inconsistent with what we observe with our *Akirin2* Emx-KO mice, and thus make the interaction between Akirin2 and RARs an unlikely mechanism for Akirin2-mediated interdigital tissue regression.

This study is the first to identify a role for Akirin2 in mammalian limb development. In limbs lacking Akirin2 in the ectoderm, we show that interdigital PCD fails and mesenchymal cells continue to proliferate, causing soft-tissue syndactyly due to retained FGF8 expression. Future work will address the protein-protein interactions of Akirin2 in limb epithelium and the specific chromatin remodelling mechanisms that are important for the downregulation of FGF8 during limb development.

## Methods

### Antibodies

Antibodies used in this manuscript are as follows: Abcam: Akirin2 (ab221475) 1:200. Cell Signaling Technology: Cleaved caspase-3 (#9661) 1:100. Clontech: DsRed (632496) 1:500. Roche: anti-digoxigenin-AP Fab fragments (11093274910) 1:2000.

### Generation of knockout mice

Details of the *Emx1-Cre* mice used for this manuscript are available in our previous study using floxed *Akirin2* knockouts^12^. Briefly, floxed *Akirin2* conditional mutant mice^11^ were crossed with a line expressing *Cre* from the *Emx-1* locus^47^ to excise the floxed *Akirin2* allele. We also crossed a tdTomato reporter, *Ai14-tdTomato* into this line, which contains a floxed stop cassette preceding the *tdTomato* gene in the *ROSA26* locus. All lines were congenic to C57BL/6 and animal experiments were approved and performed in accordance with the University of Iowa’s Institutional Animal Care and Use Committee (IACUC) and NIH guidelines. These mice are designated as Emx-KO in the manuscript and Emx1-Cre;Aki2^fl/fl^ in the figures, compared with Emx1-Cre;Aki2^fl/+^ designated as heterozygote controls. Heterozygotes showed no obvious abnormalities and appear to develop as wild-type animals do.

### RT-PCR and qPCR

Limb buds were dissected from mice at E10.5 or E14 and placed into TRIzol® (Thermo Fisher Scientific). RNA was extracted following manufacturer’s protocol and RNA cleanup was performed using the QIAGEN RNeasy Mini kit. RNA was converted to cDNA using the High Capacity cDNA reverse transcription kit (Applied Biosystems) and PCR was performed using primers designed across multiple exons and exon-exon boundaries for mouse Akirin 2 (reported previously in^12^). Akirin 2 - Exon 1- Exon 2 F 5′-CGC CTC GCC GCA GAA GTA TC-3′, R 5′-CAA CCT GGA TCT GCC TGC TGA AA-3′; Exon2/3 junction-Exon 5 F 5′-GCA TCA CCA GGG ACT TCA TCT-3′, R 5′-ACA AAG AAC AAG GCA GCC CA-3′. PCR cycling parameters for 30 cycles were: 95 °C 1 minute, 55 °C 15 seconds, 72 °C 1 minute.

For qPCR experiments, forelimbs were dissected from embryos collected at E12.5 and placed into RNAlater® (Ambion). RNA was extracted using the miRVANA RNA extraction kit (Invitrogen) and cDNA was prepared using the High Capacity cDNA reverse transcription kit (Applied Biosystems). Control and Akirin2 Emx-KO RNA was assessed for quantity using SYBR green chemistry (Roche) in a Roche LightCycler® 480 Real-Time PCR system and normalised to β-actin levels. *BMP2*, *BMP4* and sonic hedgehog (*Shh*) primers were published previously^48^. *Connexin-43* primers: F 5′-CTTTCATTGGGGGAAAGGCG-3′, R 5′-CTGGGCACCTCTCTTTCACTT-3′. *Twist1* primers were previously reported in^49^. All other primers used for qPCR were found using the PrimerBank database^50–52^ with the following PrimerBank IDs: *Runx2*, 6063419a1; *Bmpr1a*, 538363a1; *Grem1*, 215490119c1; *Fgf4*, 158508679c3; *Fgfr2*, 198594a1; *En1*, 7106305a1; *Gli3*, 120953172c1.

### Immunohistochemistry

Embryos were collected between embryonic days E10.5–E14. Embryos were fixed by immersion in 4% paraformaldehyde for 24 hours and forelimbs and hindlimbs were dissected. Samples were washed with PBS and cryoprotected with 30% sucrose, frozen in OCT and 12–18 μm cryosections were cut. Sections were blocked (2.5% BSA, 0.1% Triton-X100) for 1 hour and incubated with primary antibody diluted in blocking solution overnight at 4 °C. Sections were washed with PBS and incubated in the relevant secondary antibody conjugated to Alexa-Fluor 488 nm, 568 nm or 647 nm (Molecular Probes/Invitrogen) for 2 hours at RT, washed with PBS and counter-stained with DAPI (4′,6-diamidino-2-phenylindole) and/or SYTOX® green nucleic acid stain (Molecular probes/Invitrogen, ThermoFisher Scientific), prior to mounting with Fluoro-Gel mounting media (Electron Microscopy Services #17985-11).

### Interdigital indentation

Side-by-side images of control and Emx-KO paws were made (16x, Zeiss SteREO Discovery.V12 microscope and captured using AxioVision Rel4.8) and interdigital indentation analysis was performed according to previously published methods^53^. Briefly, a circle was drawn around each paw to best fit around digits 2–5 and a radius line drawn from the center to the outside of the circle through the interdigital web. The distance from the edge of the circle to the interdigital web (in μm) was recorded as the distance regressed. Data were collected from interdigital spaces between digits 2–3 and 3–4, with a minimum of 3 animals per genotype for each age recorded.

### Alcian Blue/Alizarin Red staining

Alcian Blue and Alizarin Red staining for gross skeletal analysis was performed on P0 and P10 Emx-KO mice (plus littermate controls) according to previously reported detailed methodology^54,55^. Briefly, mice were euthanised according to institutional IACUC protocols, skin and organs were removed, and remaining tissue fixed in several changes of 95% ethanol for up to one week. Specimens were then treated in several changes of 100% acetone for up to one week followed by staining with Alcian Blue up to 8 hours. Samples were subjected to a second round of fixation in 95% ethanol and soft tissue digestion and specimen clearing was done in 1% potassium hydroxide, up to 4 hours while monitoring for signs of disarticulation. Specimens were then exposed to freshly prepared Alizarin Red S. for up to 16 hours and stored in 100% glycerol.

### EdU injections

Pregnant dams were injected intraperitoneally at E13 or E14 with EdU (5-ethynyl-2′-deoxyuridine, Invitrogen) at a concentration of 100 μg/g, 2 hours prior to embryo collection. EdU labelling was detected using the Click-iT® EdU Alexa Fluor® 488 imaging kit (Molecular probes, Invitrogen, ThermoFisher Scientific), following manufacturer’s instructions.

### Whole mount *in situ* hybridisation

Standard whole mount *in situ* hybridisation was performed using methods modified from^56^. Fixed limb tissue was dehydrated using increasing methanol concentrations (25%, 50%, 75%, 100%) and rehydrated using the same descending methanol concentrations. Samples were washed using PBS + 0.1% Triton-x100 (Tx100), digested at RT using Proteinase K, washed in PBS + 0.1% Tx100, fixed using 4% PFA, washed in PBS + 0.1% Tx100 and placed in hybridisation solution (50% formamide, 50% 2x SSC, 6% dextran sulfate) overnight at 70 °C. Probes were diluted with salmon sperm DNA, heated to 85 °C and placed in fresh hybridisation solution with the samples overnight at 70 °C. Stringency washes were performed using 2x SSC at 65 °C, and samples were washed using PBS + 0.1% Tx100 with RNase A for 60 min at 37 °C. Further washes, blocking and primary antibody incubation was performed using the Roche DIG wash and block buffer set and anti-digoxigenin-AP antibody incubation overnight. Following antibody incubation, the tissue was extensively washed, incubated at RT in alkaline buffer (100 mM NaCl, 100 mM Tris-HCl (pH 9.5), 50 mM MgCl_2_, 1% Tween20) for 10 min and the colour reaction observed using NBT (nitro blue tetrazolium) and BCIP (5-bromo-4-chloro-3-indolyl phosphate) until sufficient colour had developed. The *Fgf8* antisense probe was a kind gift from Dr. Bernd Fritzsch, Dept. of Biology, University of Iowa, previously described^53,57^.

### Imaging

Confocal and epifluorescence imaging was conducted using a Leica SPE DM2500 Confocal Microscope and Leica Application Suite software. *In situ* hybridisation and whole mount imaging was conducted using a Zeiss SteREO Discovery.V12 microscope and captured using AxioVision Rel4.8. Images were adjusted for brightness and contrast using FIJI^58,59^ or Adobe Photoshop. Alcian Blue/Alizarin Red stained skeleton preparations were photographed in 100% glycerol using a Leica M205 FA stereoscope equipped with a Leica DFC340 FX camera.

### Analysis

For EdU and CC3 quantification, a region of interest was drawn in the distal interdigital space, corresponding to the area of increased EdU-positive cells in Emx-KO mutants or CC3 in controls. Images were thresholded using Image/J-FIJI^58,59^ and cells counted in each section. EdU and DAPI cells were counted individually from 4–6 sections per animal for each genotype, for both forelimbs and hindlimbs. At least 3 animals per genotype per age were analysed. For all quantification, unpaired t-tests (Prism software, GraphPad) were used to compare between control and knockout, with significance level p < 0.05.

## Electronic supplementary material

Supplementary Information
