# Supplementary material for: An essential role for the nuclear protein Akirin2 in mouse limb interdigital tissue regression
Source: Sci Rep. 2018 Aug 16;8:12240. doi: 10.1038/s41598-018-30801-2 (PMC6095873; doi:10.1038/s41598-018-30801-2)
Supplement: Supplementary file 1 — Supplementary Information [file 41598_2018_30801_MOESM1_ESM.pdf]

An essential role for the nuclear protein Akirin2 in mouse limb interdigital tissue regression

Peter J. Bosch, Leah C. Fuller & Joshua A. Weiner*

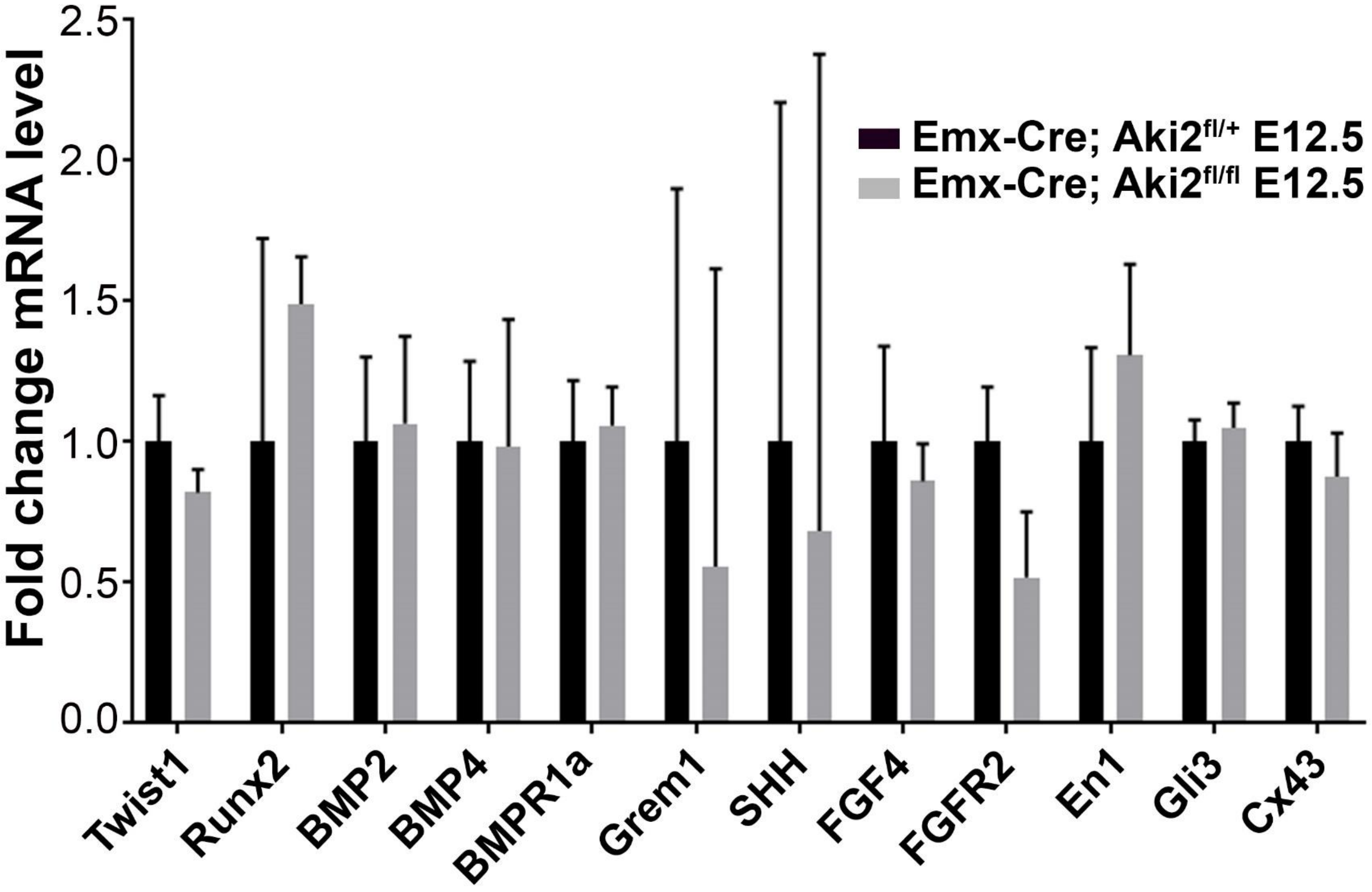

Supplementary Fig S1: Loss of Akirin2 in the limb epithelium does not significantly alter the overall expression levels of several syndactyly-associated genes. Control and Aki2 Emx-KO embryos were collected at E12.5 and forelimbs dissected and assessed for mRNA expression differences using quantitative real-time PCR (qPCR). All samples were normalised to β-actin levels. n = 6-9 Con; n = 5-7 Aki2 KO, run in triplicate wells.
